# Epidemiological Profile of Pediatric Patients with Acute Lymphoblastic Leukemia Admitted to Four Hospitals in Curitiba, Southern Brazil

**DOI:** 10.3390/medsci14020318

**Published:** 2026-06-15

**Authors:** Regiane Nogueira Spalanzani, Liana Alves de Oliveira, Sara Cristina Lobo-Alves, Thaís Muniz Vasconcelos, Luiza Souza Rodrigues, Damaris Krul, Adriele Celine Siqueira, Curitiba Transcriptomics and Microbiomics ALL Consortium, Roberto Rosati, Libera Maria Dalla-Costa, Lorena Bavia

**Affiliations:** 1 Faculdades Pequeno Príncipe, Av. Iguaçu, 333, Rebouças, Curitiba CEP 80230-020, PR, Brazil; 2 Instituto de Pesquisa Pelé Pequeno Príncipe, Av. Silva Jardim, 1632, Água Verde, Curitiba CEP 80250-060, PR, Brazilrobrosit@gmail.com (R.R.); 3 Laboratório Central do Estado do Paraná (LACEN-PR), R. Sebastiana Santana Fraga, 1395, Guatupê, São José dos Pinhais CEP 83060-500, PR, Brazil

**Keywords:** acute lymphoblastic leukemia, pediatric oncology, regional epidemiology, environmental exposure, occupational exposures, cancer treatment centers

## Abstract

Background/Objectives: Acute lymphoblastic leukemia (ALL) is the most common childhood malignancy. Understanding its epidemiological characteristics is essential for guiding public health strategies. In this study, we characterized the epidemiological profiles that may contribute to the risk of ALL in children in southern Brazil. Methods: Clinical and epidemiological data from 71 children (1–15 years old) admitted and newly diagnosed with ALL at four hospitals in Curitiba, Paraná, Brazil, were retrieved and analyzed. Results: Among the 71 children with ALL, the majority were male (*n* = 43, 60.6%), with an age range of 1–3 years (*n* = 26, 36.6%), self-identified as White (*n* = 47, 66.2%), and were born in Paraná state (*n* = 61, 85.9%). Nearly half had a family history of cancer (*n* = 33, 46.5%), primarily among grandparents (*n* = 36, 61%). Parental environmental exposures included smoking (*n* = 30, 42.3%) and occupational exposure to chemicals or radiation (*n* = 17, 23.9%). At diagnosis, most patients (*n* = 43, 60.5%) had a bone marrow blast count > 70%, and 27 patients (38%) had a peripheral blood blast count > 70%. B-cell ALL was the predominant subtype (*n* = 61, 85.9%). In B-cell ALL cases, the most frequent molecular subtype was high hyperdiploidy (*n* = 17, 23.9%). White blood cell counts differed significantly between the B-cell ALL and T-cell ALL groups (*p =* 0.029). Conclusions: Our findings provide insights into ALL epidemiology in southern Brazil and highlight regional differences across the country.

## 1. Introduction

Acute lymphoblastic leukemia (ALL) is the most common childhood cancer worldwide, representing approximately 25% of all pediatric malignancies [1]. It involves uncontrolled proliferation of immature lymphoid cells in the bone marrow, peripheral blood, and other organs, requiring prompt diagnosis and treatment [2]. ALL occurs predominantly in children aged 2–5 years, with fewer cases in infants and adolescents, and is more frequent in males [2]. Although chemotherapy has improved survival rates, it carries risks, including infections, organ toxicity, and long-term side effects [3,4]. The identification of prognostic factors like age at diagnosis, white blood cell (WBC) count, genetic alterations, and initial therapy response is crucial for treatment planning [5].

In 2021, 46,304 cases of ALL were reported in children globally, representing a 14% decrease from 1990 [2]. The National Cancer Institute reported 40 cases per million children aged 0–14 years in the United States [5]. In a Brazilian study encompassing 16 population-based cancer registries, the age-adjusted median incidence rate of leukemia among children aged 0–14 years was 53.3 cases per million, with no significant sex differences at 3 years of age [6]. The incidence of childhood leukemia in Brazil showed regional variation from 1997 to 2004 [6], with the population having unique characteristics due to interethnic relationships [7]. A study in Piaui, northeast Brazil, reported that 93.56% of childhood leukemia hospitalizations were among mixed-race individuals [8]. Most studies on pediatric ALL in Brazil have been conducted in the southeast and northeast regions. Although the exact cause is often unknown, ALL development is associated with genetic, environmental, or preexisting conditions [9].

Brazil is an expansive and highly heterogeneous country with substantial regional differences in sociodemographic and environmental factors, which may influence the epidemiological profile of cancers, such as ALL [10]. Therefore, findings from a single region may not be generalizable to the entire country. In this context, to contribute to the understanding of ALL in Brazil, we aimed to characterize the sociodemographic, epidemiological, clinical, and laboratory profiles, as well as environmental and occupational factors, of children with ALL admitted to four hospitals in Curitiba, Paraná, southern Brazil.

## 2. Methods

### 2.1. Participants

Between May 2021 and February 2024, families of 84 children newly diagnosed with ALL and admitted to four hospitals in Curitiba, Paraná, Brazil, were invited to participate in an ongoing project titled “Searching for Markers of Therapeutic Response in Acute Lymphoblastic Leukemia (ALL): Monitoring Variations in Gene Expression and Enteric Microbiome in Pediatric Patients with ALL Throughout Chemotherapy Treatment.” The inclusion criteria were pediatric patients aged 1–15 years, newly diagnosed with ALL, and without syndromes predisposing to ALL, such as Down syndrome or Fanconi anemia. Children diagnosed with B-cell acute lymphoblastic leukemia (B-ALL) or T-cell acute lymphoblastic leukemia (T-ALL) were included. Participants who withdrew at any stage of the study were excluded.

The participating institutions and number of invitations extended were as follows: Hospital Pequeno Príncipe (HPP; May 2021–February 2024, 45 invitations), Hospital Erasto Gaertner (HEG; December 2021–February 2024, 32 invitations), Complexo Hospital de Clínicas da Universidade Federal do Paraná (CHC/UFPR; March 2022–February 2024, 1 invitation), and Hospital Universitário Evangélico Mackenzie (HUEM; June 2022–February 2024, 6 invitations). This project was approved by the Research Ethics Committees of the four participating hospitals under the Certificate of Presentation for Ethical Consideration numbers: 21767019.0.0000.0097 (HPP), 21767019.0.3001.0098 (HEG), 21767019.0.3002.0096 (CHC/UFPR), and 21767019.0.3004.0103 (HUEM). Informed consent and/or assent were obtained from all participants. None of the patients had a history of primary immunodeficiency. Additionally, none of the patients received prior treatment with cytotoxic or radioactive agents or immunotherapy.

### 2.2. Data Collection and Statistical Analysis

Patients were diagnosed with B-ALL or T-ALL by the clinical staff at each participating hospital, based on clinical and laboratory findings, including leukocyte morphology, immunophenotyping, cytogenetics, and molecular genetics. Similarly, the treatment protocol—comprising the Recife Protocol for ALL (RE-LLA) [11], the Berlin–Frankfurt–Münster protocol (BFM) [12], and the 2021 Protocol of the Brazilian Cooperative Group for the Treatment of Childhood Acute Lymphoblastic Leukemia (GBTLI), an ongoing multicenter Brazilian protocol—was determined by the medical team at each hospital according to the institutional criteria. Sociodemographic, epidemiological, clinical, and laboratory data were collected from the medical records and from a survey conducted with the parents or legal guardians of the participants at the time of inclusion. Based on the WBC counts in peripheral blood at diagnosis, patients were classified into three categories: >100,000 cells/mm^3^, referred to as hyperleukocytosis [13]; 50,000–100,000 cells/mm^3^ (values > 50,000 cells/mm^3^ are considered a risk factor in some treatment protocols) [11]; and <50,000 cells/mm^3^.

Statistical analyses were exploratory and descriptive, focusing on bivariate associations between categorical variables without aiming to establish causality or estimate independent effects. Owing to the small sample sizes and sparse contingency tables, Fisher’s exact test was used to ensure robust and accurate inference. For quantitative data (age and WBC counts), groups were compared using the Mann–Whitney test. Further, because of the limited number of T-ALL cases, subgroup comparisons were considered exploratory and should be interpreted with caution. Analyses were performed using GraphPad Prism software, version 8.0.1.

## 3. Results

**Figure 1:**
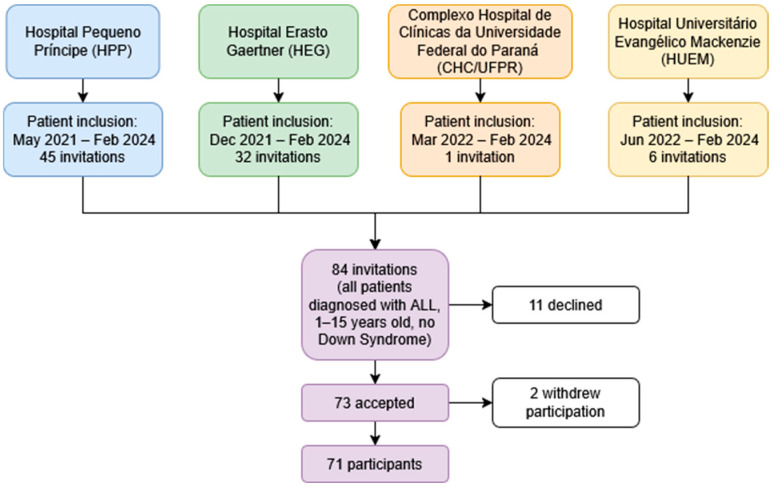
A flow diagram of a multicenter cohort of pediatric acute lymphoblastic leukemia (ALL) patients across four hospitals. Abbreviation: ALL, acute lymphoblastic leukemia. Colors represent different hospitals.

**Figure 2:**
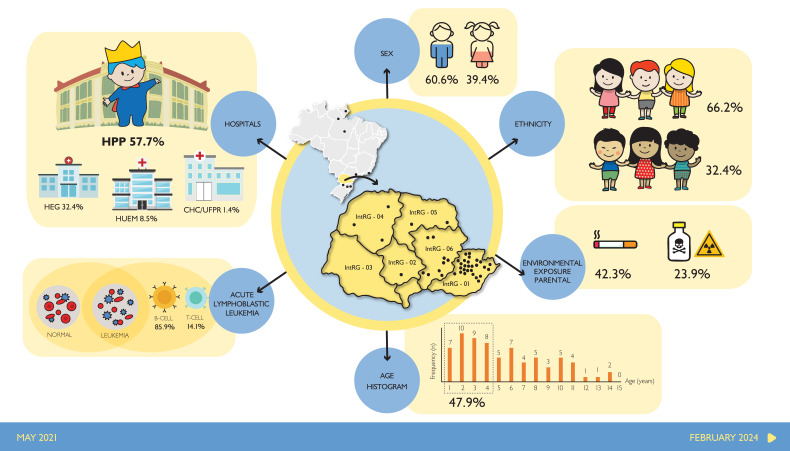
A summary of the main findings of pediatric patients with acute lymphoblastic leukemia in southern Brazil. Distribution of patients with ALL according to hospital, sex, ethnicity, parental environmental exposure, age at diagnosis, and ALL type. The map shows the geographic distribution of cases across immediate geographic regions. Abbreviations: ALL, acute lymphoblastic leukemia; HPP, Hospital Pequeno Príncipe; HEG, Hospital Erasto Gaertner; HUEM, Hospital Universitário Evangélico Mackenzie; CHC/UFPR, Complexo Hospital de Clínicas da Universidade Federal do Paraná; IntRG, intermediate region; IntRG-01, Curitiba region; IntRG-02, Guarapuava region; IntRG-03, Cascavel region; IntRG-04, Maringá region; IntRG-05, Londrina region; IntRG-06, Ponta Grossa region.

Of the 84 eligible participants, 11 refused to participate and 2 withdrew after obtaining initial consent; therefore, a total of 71 pediatric patients with ALL were evaluated (Figure 1). The sociodemographic, epidemiological, clinical, and laboratory characteristics of the participants are summarized in Table 1 and Figure 2.

**Figure 3:**
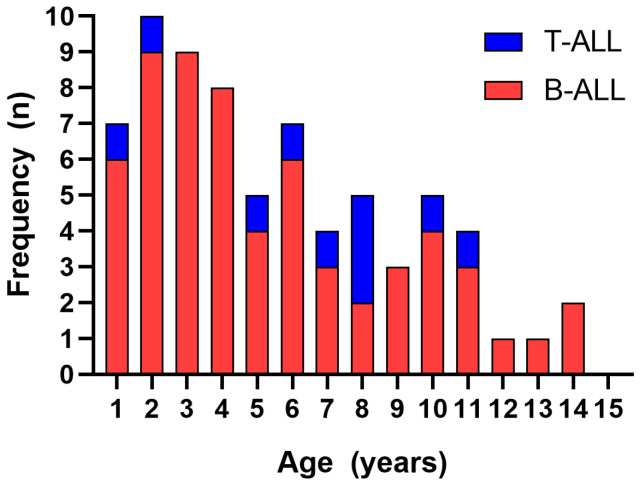
Age histogram of children with T-cell acute lymphoblastic leukemia and B-cell acute lymphoblastic leukemia admitted to four Hospitals in Curitiba, Paraná. Abbreviations: T-ALL, T-cell acute lymphoblastic leukemia; B-ALL, B-cell acute lymphoblastic leukemia.

Among the 71 pediatric patients, 61 (85.9%) had B-ALL, and 10 (14.1%) had T-ALL. Most children with ALL were male (*n* = 43, 60.6%), with no significant sex differences between the B-ALL and T-ALL groups (*p* = 0.296). Most patients were aged 1–3 years (*n* = 26, 36.6%), followed by 4–6 years (*n* = 20, 28.2%), 7–9 years (*n* = 12, 16.9%), 10–12 years (*n* = 10, 14.1%), and 13–15 years (*n* = 3, 4.2%) (Table 1). Overall, almost half of the patients (*n* = 34, 47.9%) were aged between 1 and 4 years (Figure 3). No significant differences in age distribution were observed between the B-ALL and T-ALL groups. The patients’ age varied from 1 to 14 years (mean: 5.46 ± 3.55 years, median: 5 years). Patients with B-ALL had a mean age of 5.31 ± 3.63 years and median age of 4 years. Among those with T-ALL, the maximum age was 10 years, mean age was 6.4 ± 3.03 years, and median age was 7 years. No significant difference in age was observed between the patients with B-ALL and those with T-ALL (*p* = 0.2506).

The majority of patients self-identified as White (*n* = 47, 66.2%); most children were eutrophic (*n* = 40, 56.4%) and born at full term (*n* = 57, 80.3%). No significant differences in ALL subtypes were observed when comparing White versus non-White patients, eutrophic versus non-eutrophic status, and full-term versus non–full-term births. Cesarean section was the most common delivery method, observed in 49 cases (69.0%), and 40 children (56.3%) did not receive exclusive breastfeeding during the first 6 months of life.

**Figure 4:**
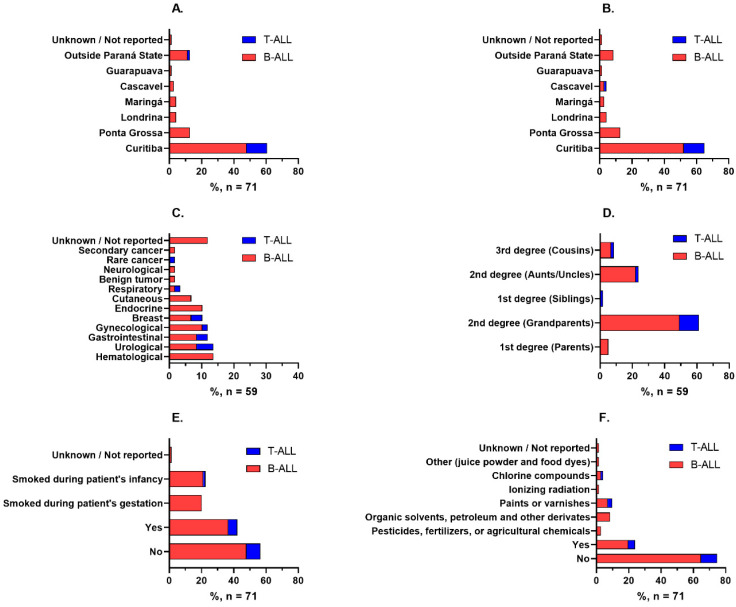
Distribution of demographics, cancer history in relatives, and environmental/ occupational exposure characteristics among patients with T-cell acute lymphoblastic leukemia and those with B-cell acute lymphoblastic leukemia. (**A**). Intermediate region of birth. (**B**). Intermediate region of residence. (**C**). Type of cancer in relatives. (**D**). Degree of kinship with affected relatives. (**E**). Parental smoking. (**F**). Parental occupational exposure to chemicals or radiation. Data are presented as percentages for each acute leukemia subtype. Additional information regarding these results is provided in Supplemental Table S1. Abbreviations: T-ALL, T-cell acute lymphoblastic leukemia; B-ALL, B-cell acute lymphoblastic leukemia.

**Figure 5:**
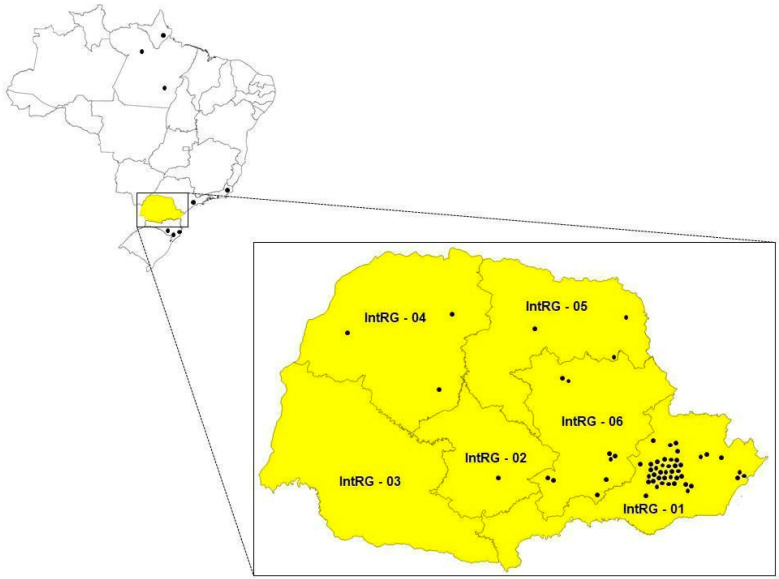
Geographical distribution of patients’ birthplaces. Map of Brazil highlighting the state of Paraná and its six intermediate geographic regions, with black dots indicating the place of birth of each patient. Abbreviations: IntRG, intermediate region; IntRG-01, Curitiba region; IntRG-02, Guarapuava region; IntRG-03, Cascavel region; IntRG-04, Maringá region; IntRG-05, Londrina region; IntRG-06, Ponta Grossa region. This figure is based on the intermediate geographic regional division of Paraná, as defined by Instituto Brasileiro de Geografia e Estatística—IBGE [16].

Most patients were born in Paraná state (61/71, 85.9%), of which 43 (70.5%) were born in the intermediate geographical region of Curitiba and 28 (65.1%) in Curitiba city (Figure 4A). The status of the patients’ residences was also assessed. Most patients (39/71, 54.9%) remained at their birth residences, whereas 42.3% (30/71) moved (Figure 4B). Among those who moved within the state of Paraná, only one changed the geographic region. The state of Paraná and its intermediate geographical regions, along with the birthplace of each patient, are shown in Figure 5.

Approximately 50% of the children with ALL (*n* = 33, 46.5%) reported having at least one relative with a history of cancer. In total, 59 cases of cancer in the family were reported by the parents or legal guardians of the participants. Fifteen (25.4%) children with ALL had at least two cases of cancer in the family. Among those with a family history, 39 (66.1%) reported cancer in the direct line of kinship and 20 (33.9%) in the collateral line, with no significant difference between the B-ALL and T-ALL groups (*p* = 0.703). Considering the degree of kinship, most reported cases of cancer occurred in grandparents of children with ALL (36, 61.0%), followed by cases in aunts/uncles (14, 23.7%). The main types of cancer in the family of children with ALL were hematological (*n* = 8, 13.6%) and urological (*n* = 8, 13.6%). Additional information on family cancer history is shown in Figure 4C,D.

Regarding environmental and occupational factors, parental occupational exposure to chemicals or radiation was reported in 17 (23.9%) patients, whereas 30 (42.3%) reported exposure to tobacco smoke. In both cases, no significant differences were observed between the leukemia subtypes (*p* = 0.696 and *p* > 0.99, respectively). Additional information on parental exposure to chemicals/radiation or parental smoking is shown in Figure 4E and 4F, respectively.

Medical records of children with ALL were analyzed to extract information about clinical findings, including organomegaly, lymphadenopathy or skin lesions, involvement of the central nervous system (CNS) and testis, blast percentages at diagnosis in peripheral blood and bone marrow, and molecular testing. The findings are presented in Table 2.

Most children with ALL were admitted to HPP (*n* = 41, 57.7%), followed by HEG (*n* = 23, 32.4%), HUEM (*n* = 6, 8.5%), and CHC/UFPR (*n* = 1, 1.4%). Regarding healthcare coverage, 48 patients (67.6%) were admitted under the Brazilian public health system and 23 (32.4%) under private health insurance. Among clinical findings, organomegaly was observed in 22 (31.0%) patients with ALL, lymphadenopathy in 19 (26.8%) patients, and skin lesions in 4 (5.6%) patients. No significant difference in these findings was observed between the B-ALL and T-ALL groups (*p* > 0.05). CNS and testicular involvement were identified in 5 (7.0%) and 1 (2.3%) patients, respectively, with no significant difference between the ALL subtypes. At diagnosis, 43 (60.5%) children with ALL had ≥70% blasts in the bone marrow, and 27 (38%) had ≥70% blasts in peripheral blood; however, no significant difference was observed between the B-ALL and T-ALL groups.

At diagnosis, most children (*n* = 59, 83.1%) had a peripheral WBC count of <50,000 cells/mm^3^, and 12 (16.9%) had counts ≥ 50,000 cells/mm^3^. The distribution of WBC counts varied significantly across ALL subtypes, with higher WBC counts observed in children with T-ALL than in those with B-ALL (*p* = 0.029). WBC counts varied from 1450 to 686,600 cells/mm^3^ (mean: 50,062 ± 118,756 cells/mm^3^, median: 8130 cells/mm^3^). B-ALL patients had a mean of 36,244 ± 102,110 cells/mm^3^ and a median of 7010 (1520–686,600) cells/mm^3^. WBC count was significantly higher in T-ALL patients, who had a mean of 134,351 ± 176,050 cells/mm^3^ and a median of 44,085 (1450–508,460) cells/mm^3^ (*p* = 0.0304).

Among children with B-ALL, molecular subtyping was not otherwise specified in 29 (40.8%) cases. Nevertheless, high hyperdiploidy (HHD) was present in 17 (23.9%) cases, followed by *ETV6::RUNX1* fusion in 12 (16.9%) cases. Less frequent molecular subtypes included *TCF3::PBX1* fusion (*n* = 2, 2.8%) and *BCR::ABL1* fusion (*n* = 1, 1.4%). Regarding ALL treatment protocols, most patients (*n* = 40, 56.3%) were assigned to the Brazilian Childhood Cooperative Group for Treatment of ALL in Children 2021 protocol, an ongoing Brazilian multicenter research protocol in which HPP and HEG are participating centers. This was followed by assignment to the Recife Protocol for ALL [11] (*n* = 20, 28.2%) and Berlin–Frankfurt–Münster protocol [12] (*n* = 11, 15.5%). Each patient’s protocol assignment was determined by the medical team of the hospital to which they were admitted according to the institutional criteria.

## 4. Discussion

ALL is the most common childhood malignancy and a significant public health concern in the pediatric population of Brazil. Considering Brazil’s continental size and regional disparities, it is essential to understand the epidemiological and clinical features of ALL in different regions. However, most studies on pediatric ALL have focused on the southeast and northeast regions, followed by the south region. In this study, we present the clinical and epidemiological findings of children diagnosed with ALL in Curitiba, Paraná, Brazil.

According to the National Cancer Institute, from 2017 to 2020, 42 cases of lymphoid leukemia were reported in Paraná in children aged 1–14 years (23 boys and 19 girls) in population-based cancer registries [17]. This number may be underestimated owing to the limitations of data collection [18]. Among the 71 pediatric patients with ALL, 14.1% had T-ALL, a proportion similar to that observed worldwide [19,20,21]. In other Brazilian studies, T-ALL accounted for 8–25% of childhood ALL cases [22,23,24].

Male sex was more prevalent in our cohort (60.6%), consistent with findings reported in Brazilian [8,22,23,25,26] and international [2,27,28,29,30] studies. In our study, the majority of admissions occurred in children aged 1–4 years, whereas in other regions of Brazil, the peak incidence of ALL was observed in patients aged 2–5 years [24,31,32] and between 0 and 4 years worldwide [33,34]. Regarding ethnicity, 66.2% of our patients self-identified as White, which is consistent with the findings of another study conducted in southern Brazil [35]. In contrast, a study from northeast Brazil reported a different ethnic profile, with 93.6% of patients self-identifying as mixed race [8], which may reflect regional demographic variations within Brazil. Additionally, 21% of our patients (*n* = 15) were obese; obesity has been associated with poor survival outcomes [36].

As expected, most patients in our study were born in the state of Paraná, considering that the hospitals were located in Curitiba, the state capital. Paraná has 24 high-complexity cancer treatment centers distributed across 15 municipalities [37]. Additionally, these institutions receive pediatric patients from outside the state [17,37]. The high number of patients from HPP may reflect the longer recruitment period at this site (33 months) than at other hospitals (HEG, 27 months; HUEM, 20 months; CHC/UFPR, 24 months). Additionally, most patients (48, 67.6%) in this study were managed within the public health network Sistema Único de Saúde (Brazilian Unified Health System), which refers patients to hospitals according to regionalized care guidelines [38].

Our study showed that most patients underwent full-term delivery, with cesarean section being the most frequent procedure. In Brazil, this procedure is common, and the proportion found in our study reflects the national pattern [39]. More than half of the patients (56.3%) were not exclusively breastfed until 6 months of age. Breastfeeding, especially for ≥6 months, is associated with a low risk of ALL. It provides nutritional support, maternal antibodies, anti-inflammatory molecules, and beneficial microbes that support the infant gut microbiome, potentially modulating the immune system [40,41,42].

Almost half of the patients reported at least one case of cancer in their family, mainly among second-degree relatives (grandparents), with hematological and urological cancers being the most frequently reported in our cohort. Hematological malignancies in first- or second-degree relatives are associated with a slightly increased risk of childhood ALL [43], and other studies have reported associations with specific cancers in first-degree relatives, including testicular cancer in fathers [44], as well as esophageal, breast, prostate, connective tissue cancers, and leukemia [45]. However, some studies found no significant associations [46,47]. Overall, these findings suggest that the role of family history of cancer in childhood ALL remains controversial, highlighting the complexity of its interpretation in pediatric patients.

Parental occupational exposure to radiation and/or chemicals was reported by almost a quarter of the legal guardians of 17 (23.9%) children with ALL in our cohort. Chemicals are well-established risk factors for cancer, and hydrocarbons are of particular concern because of their widespread use in household and industrial products [48]. In our study, the most frequent parental occupational exposures were paints or varnishes (9.8%) and organic solvents (including petroleum and its derivatives) (8.5%). Exposure to pesticides, fertilizers, other agricultural products (2.8%), chlorine compounds (2.8%), and ionizing radiation (1.4%) was also reported. Associations between parental exposure to paints and pigments and childhood ALL have been previously reported [48]. Household paint exposure increases the risk of childhood ALL, particularly when it occurs shortly before conception, during pregnancy, or after birth [49,50]. In pooled analyses of 12 international case–control studies, paternal occupational pesticide exposure around conception increased the risk of childhood ALL by approximately 20%, whereas the results for maternal exposure showed inconsistent findings [50,51,52,53,54]. Only ionizing radiation has been associated with childhood ALL, and maternal exposure to X-rays during pregnancy increases the risk of leukemia in the offspring by approximately 50% [48,55,56]. Regarding parental smoking, epidemiological evidence has shown no association between maternal smoking and the risk of childhood ALL and only a weak association between paternal smoking and the risk of childhood ALL [9,57,58,59].

Physical examination at diagnosis revealed organomegaly (31.8%) and lymphadenopathy (26.9%) in children with ALL. In a study from Pakistan [60], almost 65% of children diagnosed with ALL had physical abnormalities, including hepatomegaly and splenomegaly. Similarly, in Mexico [61], organomegaly was reported in >60% of cases. Similar findings were observed in Ceará, northeastern Brazil [25]. Organomegaly and lymphadenopathy are common in pediatric patients with ALL, although their prevalence varies regionally. Skin manifestations were observed in 4 (5.6%) children with ALL in our study, which typically indicates advanced disease with extramedullary infiltration and is often associated with a poor prognosis [62]. In our study, 5 (7.0%) children with ALL were diagnosed with CNS involvement, a condition that can be detected in 3–20% of patients [33,63,64]. Although effective treatment options are available, CNS involvement at diagnosis remains a major challenge in the management of pediatric ALL. Intensive CNS-directed treatment is nonspecific and associated with the risks of neurotoxicity, relapse, and long-term neurocognitive sequelae [65,66].

At diagnosis, a total of 12 (16.9%) children had a WBC count >50,000 cells/mm^3^, of which 8 (11.3%) had hyperleukocytosis (>100,000 cells/mm^3^). In Rio de Janeiro, southeast Brazil, Bonilha et al. found that 38.7% of children with ALL had WBC counts >50,000 cells/mm at diagnosis, and 20.4% of them had hyperleukocytosis [22]. Similarly, in Bahia, northeast Brazil, 25.6% of patients had WBC counts >50,000 cells/mm at diagnosis, of which half (12.8%) had hyperleukocytosis [23]. Children with hyperleukocytosis at ALL diagnosis were previously observed, varying from 11.2% [67] to 22.5% [68] in Europe. An elevated WBC count at diagnosis is associated with poor prognosis in children with ALL [69]. In this study, although a significant difference was observed in the WBC counts between patients with B-ALL and those with T-ALL, it should be interpreted with caution because of the limited number of T-ALL cases. Half of the children with T-ALL (*n* = 5, 50%) had elevated WBC counts, and 40% had counts >100,000 cells/mm^3^. Similar findings regarding elevated WBC counts in patients with T-ALL have been reported in Poland [70]. In the study, the authors also observed a correlation between high WBC count at diagnosis and high incidence of infection as a complication of ALL therapy. Most epidemiological studies on childhood ALL, particularly in Brazil, do not stratify clinical or laboratory variables by immunophenotype, thereby limiting the detailed comparisons.

Among patients with B-ALL, HHD (23.9%) was the most frequent molecular subtype, followed by *ETV6*::*RUNX1* (16.9%). These are the most common subtypes in children with B-ALL worldwide, with HHD and *ETV6*::*RUNX1* frequencies varying from 15% to 40% and from 13% to 34%, respectively [19,33,34]. In previous studies in Brazil [22,23], a low frequency of HHD was observed (3–6%), but a large proportion of patients in those studies had no cytogenetic results available, whereas in this study, karyotyping results were available for 70% of patients with B-ALL. In a study conducted in Rio de Janeiro [22], *BCR*::*ABL1* was the most frequently detected subtype (15.4%). This gene fusion is usually observed in 2–8% of pediatric B-ALL cases [34,45] and was observed in only one patient in the present study and in another Brazilian study [23].

This study has some limitations. First, the absence of a control group prevented comparisons between environmental exposures and other potential risk factors. Second, the relatively small number of patients may have limited the generalizability of our findings. Finally, the information was collected only at the time of diagnosis, which may have restricted the assessment of exposure and clinical characteristics over time.

## 5. Conclusions

This multicenter study describes the demographic, clinical, and environmental characteristics of pediatric patients with ALL from four hospitals in Curitiba, southern Brazil. The cohort was characterized by a predominance of male patients, diagnosis at an early age (1–4 years), and B-ALL subtype. Parental reports indicated environmental exposures, with tobacco use being the most frequently mentioned. In addition, nearly half of the patients had a family history of cancer, mainly among second-degree relatives, with hematological and urological malignancies being the most commonly reported. These findings highlight the importance of broader nationwide data collection and further studies to improve the understanding of childhood ALL and to support public health strategies tailored to regional contexts.

## Figures and Tables

**Table 1:** Sociodemographic and epidemiological characteristics of children with acute lymphoblastic leukemia admitted to four Hospitals in Curitiba, Paraná.

	Total *n* (%)	B-ALL *n* (%)	T-ALL *n* (%)	*p* Value
**Total number of patients**	71 (100)	61 (85.9)	10 (14.1)	-
**Sex**				
Male	43 (60.6)	35 (57.4)	8 (80.0)	0.296
Female	28 (39.4)	26 (42.6)	2 (20.0)	
**Age (years)**				
1 to 3	26 (36.6)	24 (39.3)	2 (20.0)	0.245
4 to 6	20 (28.2)	18 (29.5)	2 (20.0)	
7 to 9	12 (16.9)	8 (13.1)	4 (40.0)	
10 to 12	10 (14.1)	8 (13.1)	2 (20.0)	
13 to 15	3 (4.2)	3 (5.0)	-	
Ethnicity *				
White	47 (66.2)	41 (67.2)	6 (60.0)	0.719
Non-white	23 (32.4)	19 (31.1)	4 (40.0)	
*Mixed-race*	18 (25.4)	16 (26.2)	2 (20.0)	-
*Black*	3 (4.2)	2 (3.3)	1 (10.0)	-
*East Asian*	1 (1.4)	1 (1.6)	-	-
*Indigenous*	1 (1.4)	-	1 (10.0)	-
Not declared	1 (1.4)	11 (1.6)	-	-
Nutritional status **				
Eutrophic	40 (56.4)	37 (60.7)	3 (30.0)	0.678
Non-eutrophic	31 (43.6)	24 (39.3)	7 (70.0)	
*Obese*	15 (21.1)	11 (18.0)	4 (40.0)	-
*Overweight*	15 (21.1)	12 (19.7)	3 (30.0)	-
*Underweight*	1 (1.4)	1 (1.6)	-	-
**State of birth**				
Paraná State	61 (85.9)	52 (85.2)	9 (90.0)	-
Other Brazilian State	9 (12.7)	8 (13.1)	1 (10.0)	-
Unknown/Not reported	1 (1.4)	1 (1.6)	-	-
**State of residence**				
Paraná State	64 (90.1)	54 (88.5)	10 (100.0)	-
Other Brazilian State	6 (8.5)	6 (9.8)	-	-
Unknown/Not reported	1 (1.4)	1 (1.6)	-	-
**Gestational time**				
Full-term delivery (between 37 and 41 weeks)	57 (80.3)	50 (82.0)	7 (70.0)	0.163
Other than full-term delivery	10 (14.1)	7 (11.5)	3 (30.0)	
*Preterm delivery (less than 37 weeks)*	4 (5.6)	4 (6.6)	-	-
*Post-term delivery (over 41 weeks)*	6 (8.5)	3 (4.9)	3 (30.0)	-
Unknown/Not reported	4 (5.6)	4 (6.6)	-	-
**Type of birth delivery**				
Cesarean (C-section)	49 (69.0)	42 (68.9)	7 (70.0)	>0.99
Vaginal	21 (29.6)	18 (29.5)	3 (30.0)	
Unknown/Not reported	1 (1.4)	1 (1.6)	-	-
**Exclusive breastfeeding until 6 months**				
No	40 (56.3)	36 (59.0)	4 (40.0)	0.301
Yes	29 (40.8)	23 (37.7)	6 (60.0)	
Unknown/Not reported	2 (2.8)	2 (3.3)	-	-
**Family history of cancer**				
No	34 (47.9)	29 (47.5)	5 (50.0)	>0.99
Yes	33 (46.5)	29 (47.5)	4 (40.0)	
Unknown/Not reported	4 (5.6)	3 (4.9)	1 (10.0)	-
Number of cases of cancer in patients’ families ***				
Total number of reported cases	59 (-)	49 (80.3)	10(100.0)	-
One case	14 (48.3)	13 (21.3)	1 (10.0)	0.615
Two cases	15 (51.7)	13 (21.3)	2 (20.0)	
Three to five cases	4 (5.6)	3 (4.9)	1 (10.0)	
History of cancer in patients’ family and degrees of kinship ***				
Direct line	39 (66.1)	32 (52.5)	7 (70.0)	0.703
Collateral line	20 (33.9)	17 (27.9)	3 (30.0)	
**Parental occupational exposure to chemicals or radiation**				
No	53(74.7)	46 (75.4)	7 (70.0)	0.696
Yes	17(23.9)	14(23.0)	3 (30.0)	
Unknown/Not reported	1 (1.4)	1 (1.6)	-	-
**Parental smoking exposure**				
No	40 (56.3)	34 (55.7)	6 (60.0)	>0.99
Yes	30 (42.3)	26 (42.6)	4 (40.0)	
Unknown/Not reported	1 (1.4)	1 (1.6)	-	-

* Ethnicity was classified according to the Instituto Brasileiro de Geografia e Estatística—IBGE [14]. ** Nutritional status was calculated using body mass index for age z-score according to the World Health Organization classification [15]. *** Refers to the 33 patients with a family history of cancer; multiple cancers in one individual were counted separately. Abbreviations: ALL, acute lymphoblastic leukemia. Bold information represents classes of parameters. Italicized information represents subcategories within the non-italicized above category (not considered in the statistical analysis).

**Table 2:** Clinical and laboratory data of children with acute lymphoblastic leukemia.

	Total *n* (%)	B-ALL *n* (%)	T-ALL *n* (%)	*p* Value
**Total number of patients**	71 (100)	61 (85.9)	10 (14.1)	-
**Admission hospital**				
HPP	41 (57.7)	37 (60.7)	4 (40.0)	-
HEG	23 (32.4)	18 (29.5)	5 (50.0)	-
HUEM	6 (8.5)	5 (8.2)	1 (10.0)	-
HC/UFPR	1 (1.4)	1 (1.6)	-	-
**Healthcare coverage**				
Public	48 (67.6)	42 (68.9)	6 (60.0)	-
Private	23 (32.4)	19 (31.1)	4 (40.0)	-
**Clinical findings**				
Organomegaly	22 (31.0)	15 (24.6)	7 (70.0)	0.912
Lymphadenopathy	19 (26.8)	14 (23.0)	5 (50.0)	
Skin lesions	4 (5.6)	3 (4.9)	1 (10.0)	
**CNS involvement**				
No	66 (93.0)	57 (93.4)	9 (90.0)	0.543
Yes	5 (7.0)	4 (6.6)	1 (10.0)	
**Testicular involvement**				
No	42 (97.7)	34 (97.1)	8 (100.0)	>0.99
Yes	1 (2.3)	1 (2.9)	-	
**Blasts in BM**				
Less than 70%	19 (26.8)	17 (27.9)	2 (20.0)	>0.99
Over than 70%	43 (60.5)	38 (62.3)	5 (50.0)	
Test not performed *	9 (12.7)	6 (9.8)	3 (30.0)	-
**Blasts in peripheral blood**				
Less than 70%	44 (62.0)	38 (62.3)	6 (60.0)	>0.99
Over than 70%	27 (38.0)	23 (37.7)	4 (40.0)	
**WBC count in peripheral blood (cells/mm^3^)**				
<50,000	59 (83.1)	54 (88.5)	5 (50.0)	0.029
≥50,000 to <100,000	4 (5.6)	3 (4.9)	1 (10.0)	
≥100,000	8 (11.3)	4 (6.6)	4 (40.0)	
**Molecular testing done**				
PCR	56 (78.9)	48 (78.7)	8 (80.0)	-
FISH	48 (67.6)	45 (73.8)	3 (30.0)	-
NGS	2 (2.8)	1 (1.6)	1 (10.0)	-
None of the above	2 (2.8)	2 (3.3)	0 (0.0)	-
**Genetic alteration tested by PCR or FISH**				
*BCR::ABL1*	66 (93.0)	57 (93.4)	9 (90.0)	-
*ETV6* *::RUNX1*	58 (81.7)	52 (85.2)	6 (60.0)	-
*TCF3::PBX1*	42 (59.2)	38 (62.3)	4 (40.0)	-
*KMT2Ar*	57 (80.3)	51 (83.6)	6 (60.0)	-
Chromosome counting	15 (21.1)	13 (21.3)	2 (20.0)	-
**Karyotype testing**				
At least 20 metaphases analyzed	16 (22.5)	15 (24.6)	1 (10.0)	-
Less than 20 metaphases analyzed	32 (45.1)	28 (45.9)	4 (40.0)	-
No results **	20 (28.2)	16 (26.2)	4 (40.0)	-
Test not performed *	3 (4.2)	2 (3.3)	1 (10.0)	-
**Molecular subtype**				
B-ALL NOS	29 (40.8)	29 (47.5)	-	-
HHD	17 (23.9)	17 (27.9)	-	-
*ETV6*::*RUNX1*	12 (16.9)	12 (19.7)	-	-
*TCF3*::*PBX1*	2 (2.8)	2 (3.3)	-	-
*BCR*::*ABL1*	1 (1.4)	1 (1.6)	-	-
T-ALL	10 (14.1)	-	10 (100.0)	-
ALL treatment protocol ***				
GBTLI	40 (56.3)	35 (57.4)	5 (50.0)	-
RE-LLA	20 (28.2)	16 (26.2)	4 (40.0)	-
BFM	11 (15.5)	10 (16.4)	1 (10.0)	-

* Test reported as not performed because of sample collection failure or insufficient material. ** Results not available. *** Protocol assignment was defined by clinical staff from each hospital. Abbreviations: ALL, acute lymphoblastic leukemia; HPP, Hospital Pequeno Príncipe; HEG, Hospital Erasto Gaertner; HUEM, Hospital Universitário Evangélico Mackenzie; CHC/UFPR, Complexo Hospital de Clínicas da Universidade Federal do Paraná; CNS, central nervous system; BM, bone marrow; WBC, white blood cell; HHD, high hyperdiploidy; PCR, polymerase chain reaction; FISH, fluorescent in situ hybridization; NGS, next generation sequencing; HHD, high hyperdiploidy; NOS, not otherwise specified; NA, not applicable; GBTLI, Brazilian Childhood Cooperative Group for Treatment of Acute Lymphoblastic Leukemia in Children protocol; RE-LLA, Recife Protocol for Acute Lymphoblastic Leukemia; BFM, Berlin–Frankfurt–Münster protocol. Bold information represents classes of parameters.

## Data Availability

The data presented in this study are available on request from the corresponding author. The data are not publicly available due to ethical reasons.

## Supplementary Materials

The following supporting information can be downloaded at https://www.mdpi.com/article/10.3390/medsci14020318/s1. Table S1: Detailed data underlying Figure 4. Legend: Information is organized into four worksheets containing distributions of patients according to (1) intermediate geographic region at birth and of residence, (2) family history of cancer by degree of kinship, (3) parental tobacco exposure, and (4) parental occupational exposure.

## Abbreviations

ALL	Acute lymphoblastic leukemia
HPP	Hospital Pequeno Príncipe
HEG	Hospital Erasto Gaertner
CHC/UFPR	Complexo Hospital de Clínicas da Universidade Federal do Paraná
HUEM	Hospital Universitário Evangélico Mackenzie
B-ALL	B-cell acute lymphoblastic leukemia
T-ALL	T-cell acute lymphoblastic leukemia
CNS	Central nervous system
HHD	High hyperdiploidy
INCA	Instituto Nacional de Câncer
IBGE	Instituto Brasileiro de Geografia e Estatística
WBC	White blood cell
PCR	Polymerase chain reaction
FISH	Fluorescent in situ hybridization
